# Annotations

**Published:** 1911-05-27

**Authors:** 


### Annotations.

### An Outbreak of Typhus Fever.

Typhus fever is nowadays one of those extremely rare diseases which few general practitioners have ever met with; and not infrequently, as a natural result, when it does break out, it goes for a time unrecognised. One of the ambitions of the really keen hospital resident medical officer is to detect in the casualty room a case of this banished disease, but few of them ever attain that ambition. Cases of infective endocarditis are sometimes responsible for quite a flutter of excitement in the resident's room, but it is only rarely that the excitement is found to be justified by the facts, for when all is said and done, typhus is all but an extinct disease in this country. The city of Carlisle appears to be one of the few places in England where cases of typhus still occasionally turn up; thus, in 1905 sixteen cases were discovered, though one has to go back to 1874 for a really serious epidemic. In the *Edinburgh Medical Journal* Mr. Beard gives some very interesting details of the last appearance of the fever, when there were six cases, all apparently derived from an unsuspected fatal case in an old woman who had acquired the disease from some quite unknown source. The author discusses the ætiology of typhus, and is inclined to favour vermin as potential carriers of the infection from one patient to another. He does not, however, exclude the possibility of atmospheric contamination in transmitting the disease, and of infection by exhalations, and excretions. He also points out that the common mistake in diagnosis is to report typhus as scarlet fever. The article contains many practical hints, and should be studied by every medical man whose professional lot is cast in the slums of our great cities.

### The First Medical Degree in China.

If rumour is to be believed the Chinese Government is quickly adopting Western methods and habits, the latest of which, according to the *Times*, is that of granting medical degrees. For the first time in history, we learn the Chinese Government has conferred a degree in medicine. Twenty-one candidates who have been studying at the Union Medical College, Pekin, since its foundation five years ago, presented themselves at the recent examination and sixteen of them were adjudged fully qualified to practise. This must be a very gratifying result to the college authorities, including Dr. Herbert Wenham, F.R.C.S., who thereby receive the first-fruits of their labours. Exactly how far the Government degree will have value in the eyes of the Chinese people it is impossible to say. Hitherto authorities on China have united in the opinion, recently impressed on many tired diplomatists, that the Government of China is a force and personality apart from, and disregarded by, the Chinese population. Nevertheless an enterprising beginning has been made.

### Tight Neck-clothing.

Elsewhere in this issue we publish an article from the pen of Dr. Walter G. Walford, who has been indefatigable in drawing attention to the evils which result from the wearing of tightly fitting neck-clothing. Dr. Walford has been good enough to supply us with a large number of cases in which the simple method he describes has been tried by patients suffering from such a syndrome as he himself experienced three years ago, with the best results. He is himself an excellent advertisement for the method, and it is hard to realise that so vigorous and healthy a man as he now is suffered but three years ago from what, in the opinion of a well-known specialist, was a very serious cerebral condition. There is naturally a tendency to disregard the commonplace, the obvious. And yet, as Pirogoff, in his classical essay on "Luck in Surgery" reminded us, it is just attention to the commonplace, to the little things that matter so infinitely in the attempt to obtain perfection, that the true physician or surgeon shows his greatness. We therefore sincerely commend Dr. Walford's little sermon to our readers in the hope that it will induce them at once to investigate the condition of their collars, an investigation which, we may add, was in our own case the immediate result of Dr. Walford's visit to this office and his demonstration of the fact that the editorial neck-clothing, which we had always regarded as quite loose enough, was by no means so scientifically adjusted as it ought to have been!

### The Cromer Episode.

On May 19th, at the general meeting of the society, a narrow majority passed a motion requesting Lord Cromer to resign the Vice-Presidency of the Royal Society for the Prevention of Cruelty to Animals because he is the President of the Research Defence Society. Lord Cromer took such a personal and helpful interest in the work of putting down cruelty to animals in Egypt that he was especially invited to accept the Vice-Presidency of the Royal Society for the Prevention of Cruelty to Animals, and his reputation is too firm for petty spite to detract from it. Upon the society itself the result of the meeting referred to can be nothing but disastrous. Several attempts have already been made to capture this organisation in the anti-vivisection interest; apparently for the time being this has been done, unless (as is quite possible) this is a mere snatch vote. At the same meeting similar votes concerning Lord Cheylesmore and Mr. Stockman were rejected only by the narrowest majorities. The only comfort to be drawn from the incidents is that apparently the anti-vivisectors are finding the Research Defence Society too strong for them, and that their ebullition proceeds from a consciousness that the public is beginning to discriminate as to who are the true benefactors of mankind, scientific researchers or those who would compel them to cease from a particular branch of their work.

